# Development and Characterization of Nanoemulsions for Ophthalmic Applications: Role of Cationic Surfactants

**DOI:** 10.3390/ma14247541

**Published:** 2021-12-08

**Authors:** Ana R. Fernandes, Elena Sanchez-Lopez, Tiago dos Santos, Maria L. Garcia, Amelia M. Silva, Eliana B. Souto

**Affiliations:** 1 Institute for Research & Innovation in Health, University of Porto, R. Alfredo Allen 208, 4200-135 Porto, Portugal; anaritavfernandes@gmail.com (A.R.F.); tiago.f.santos@ineb.up.pt (T.d.S.); 2 Biomedical Engineering National Institute, University of Porto, Alfredo Allen 208, 4200-135 Porto, Portugal; 3 Faculty of Engineering, University of Porto, R. Dr. Roberto Frias, 4200-465 Porto, Portugal; 4 Centre for Research and Technology of Agro-Environmental and Biological Sciences (CITAB), University of Trás-os-Montes e Alto Douro (UTAD), Quinta de Prados, 5001-801 Vila Real, Portugal; 5 Department of Pharmacy, Pharmaceutical Technology and Physical Chemistry, Faculty of Pharmacy, University of Barcelona, 08028 Barcelona, Spain; esanchezlopez@ub.edu (E.S.-L.); marisagarcia@ub.edu (M.L.G.); 6 Institute of Nanoscience and Nanotechnology (IN2UB), University of Barcelona, 08028 Barcelona, Spain; 7 Department of Biology and Environment, University of Traás-os-Montes e Alto Douro (UTAD), Quinta de Prados, 5001-801 Vila Real, Portugal; 8 Centre of Biological Engineering, Campus de Gualtar, University of Minho, 4710-057 Braga, Portugal

**Keywords:** nanoemulsions, ocular barriers, ocular delivery, cationic surfactants

## Abstract

The eye is a very complex organ comprising several physiological and physical barriers that compromise drug absorption into deeper layers. Nanoemulsions are promising delivery systems to be used in ocular drug delivery due to their innumerous advantages, such as high retention time onto the site of application and the modified release profile of loaded drugs, thereby contributing to increasing the bioavailability of drugs for the treatment of eye diseases, in particular those affecting the posterior segment. In this review, we address the main factors that govern the development of a suitable nanoemulsion formulation for eye administration to increase the patient’s compliance to the treatment. Appropriate lipid composition and type of surfactants (with a special emphasis on cationic compounds) are discussed, together with manufacturing techniques and characterization methods that are instrumental for the development of appropriate ophthalmic nanoemulsions.

## 1. Introduction

Emulsions are a mixture of two immiscible liquids in which the droplets of one liquid (dispersed phase) are dispersed in another liquid (continuous phase) stabilized by a surfactant at the interfacial boundary. Emulsions can be classified into two categories, namely, single emulsions and double emulsions. Single emulsions can be oil-in-water (o/w) when an organic phase (frequently an oily phase) is dispersed in water and water-in-oil (w/o) when water droplets are dispersed in an oil. These emulsions are formed by a single-step method [1]. Double emulsions can be water/oil/water (w/o/w) or oil/water/oil (o/w/o) and are prepared applying a two-step procedure. In o/w/o emulsion, the inner o/w pre-emulsion is suspended in an oil phase. As emulsions are formed by two immiscible liquids, they are kinetically stable. Microemulsions are thermodynamically stable and remain kinetically stable indefinitely if the initial conditions are not changed over time. Nanoemulsions (NEs) are a thermodynamically unstable system that probably will break down over time depending on the energy barriers that exist between the NEs and the separated states of the systems. The free energy of the systems gives information about the kinetic stability of the NEs, i.e., NEs will remain stable with higher free energy. The lower free energy of the system promotes the separation of phases in NEs, which makes them highly unstable [2]. Surfactants and emulsifiers are essential to stabilize emulsions and give them a longer shelf life by sitting at the interface of oil droplets and water to stabilize the emulsion through steric stabilization or electrostatic repulsion. Emulsifiers and surfactants are included in surfactant categories, aiming to reduce the interfacial tension between the two phases of emulsions. Emulsifiers commonly used to prepare o/w emulsions have a hydrophilic–lipophilic balance (HLB) between 8 and 16. To prepare w/o emulsions, emulsifiers with HLB values around 4.7 to 6.7 are required. HLB stands for the affinity of a molecule to the different phases, i.e., oil and water [3]. 

NEs are also known as mini-emulsions and are a suitable candidate to deliver hydrophobic drugs [4,5]. NEs are emulsions with droplet sizes between 20 and 500 nm with spherical shapes [6]. NEs exhibit properties that may contribute to improve the pharmacological activity of drugs and important rheological properties [7]. The response to the mechanical deformation of NEs allows them to be transformed from viscous to elastic with the decrease in droplet size [8]. NEs are widely used in biomedical application whether to create formulations for hydrophobic drugs or in the synthesis of advanced materials. The hydrophobic drug is dissolved in the oil phase of o/w NEs, and the choice of oil phases, as well as surfactants and formulation composition, is essential to achieve optimal NEs [9]. The physicochemical properties of excipients composing the NEs are instrumental for the stability and sustained drug release over time. NEs can be used for different routes of administrations, i.e., topical, ocular, intravenous, and intranasal [10,11,12]. In this review, we give special emphasis to NEs aimed for eye administration and their main features to overcome ocular barriers. 

## 2. Ocular Barriers

**Figure 1:**
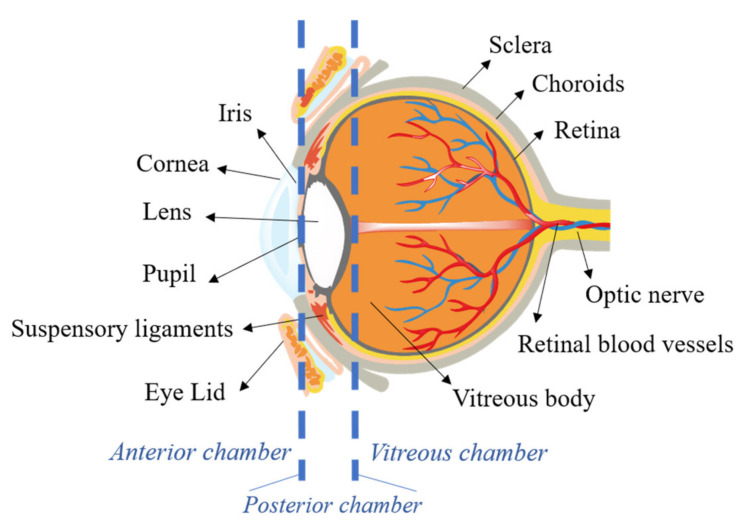
Schematic representation of the eye anatomy.

The eyes are a special organ with complex physiology, while many of their associated disorders are correlated to blindness. The structure of the eye can be divided into two segments: the anterior and posterior segments (Figure 1). The cornea, conjunctiva, aqueous humor, ciliary body, iris and crystalline lens are the elements present in the anterior segment, which occupy almost one-third of the eye. The sclera, choroid, Bruch’s membrane, the retinal pigment epithelium (RPE), retina, and vitreous humor are the remaining portion of the eye [13,14].

The cornea is the most sensitive tissue in the human body; it is transparent, avascular, thin, and highly innervated. Its thickness suffers an enhancement from the center to the periphery of cornea [15]. The cornea has innumerous nerve endings (i.e., sensory nerves, sympathetic autonomic nerve fibers, and long ciliary nerves); i.e., it is highly innervated with a higher nerve density compared to skin [16]. If any damage to the corneal epithelium occurs, it may provoke the exposure of nerve endings to the environmental surroundings, which may result in severe pain [17]. The healthy cornea is considered avascular because it does not have a supply of blood vessels; however, the epithelial and endothelial cells that constitute the cornea are metabolically active and are involved in wound ocular healing [15]. The aqueous humor is responsible for the supply of glucose and oxygen, which are both essential to maintain the normal function of the cornea [18]. The corneal epithelium, stroma, endothelium, and Bowman, Dua, and Descemet layers are the differentiated structures of the eye [19]. The most superficial epithelial cells layer is renewed by pluripotent stem cells that are present in the space between the cornea and sclera, usually occurring every 7 to 10 days. The replaced cells are eliminated into the tear film after breaking the tight junctions between them. In order to keep the tight junctions, the newer cells have the help of wing cells, which make lateral junctions between those cells [20,21].

The conjunctiva secretes mucus (that form the tissue) in the inner lining of eyelids (both upper and lower eyelids); it is a thin, semi-transparent, and high vascularized tissue [22]. The conjunctiva has efferent, afferent, and sensory nerves, making this tissue highly vascularized. Furthermore, the conjunctiva is supplied by lymphoid tissues, and histologically, it is formed by a multilayered epithelium in the superficial and a layer of stroma underlying [23,24].

The aqueous humor is an ocular fluid that is optically clear and with alkaline nature. This fluid is continuously formed by epithelial cells of ciliary body achieving almost 2.5 L/min in humans [25]. The formation of aqueous humor is achieved by diffusion, ultrafiltration, and active secretion from the plasma and is responsible for supplying nutrients and oxygen to the cornea and lens (the avascular tissues of the anterior chamber of the eye). The regulation of aqueous humor is important to regulate the ocular pressure of the eye. The iris is located behind the cornea and is considered an extension of the ciliary body. It is formed by the endothelium, epithelium and stoma. The pupil is a small opening in the lens made by the iris to regulate the quantity of light that penetrates the retina. The ciliary body is located anterior to the iris and has important functions, e.g., the secretion of aqueous humor; it helps to drain the aqueous humor and contains muscle that helps to adjust the crystalline lens to focus objects or images. The lens is another avascular structure of the eye, non-innervated, transparent, and with a biconvex form that is behind the iris [26]. The posterior part of the lens is filled with vitreous humor and the anterior part is filled with aqueous humor. The lens capsule is a barrier that regulates the passive changes of metabolites and some waste by diffusion [27].

In the posterior chamber of the eye, the sclera that is known as ‘the white part of the eye’ is an avascular, elastic, and tough structure positioned below the conjunctiva [28]. The optic nerve exists through this dense structure. The sclera is the outer of the eyeball. It frequently suffers alterations due to the external environment, namely, in the intraocular pressure [29].

The choroid is highly vascularized and innervated, and it is positioned between the peripheral sclera and the inner retinal pigment epithelium (RPE). This tissue supplies nutrients to the retina and is responsible for maintaining the volume and temperature of the eye. Histologically, the choroid is constituted by four layers, the suprachoroidea, the large-vessel layer, the intermediate-vessel layer, and the choroiocapillaris [30]. 

The RPE is formed by indivisible cells that spontaneously form a monolayer above the neural retina. In specific pathological conditions, these cells may proliferate despite their indivisible nature. This epithelium gives protection to the inner tissues, and it is responsible for the secretion of vascular endothelial growth factors and ciliary neurotropic factors, keeping then the ocular immunity and protecting the eye against oxidative damage [31,32]. The exposure to light and metabolic activity induces a highly oxidative environment in the eye. The exposure to ultraviolet radiation results in the formation of reactive oxygen species. The eye has an organized complex protection system against the oxidative damage, the pigments of the eye are capable of absorbing and filtering the light and then preventing the free radical yield, the enzymatic or non-enzymatic antioxidants act in the detoxification of free radicals, and the eye has a repairment system for the oxidized biomolecules [33].

The macula, optic disc, fovea, and the peripheral retina are constituents of the retina. The choroidal circulation is responsible for the blood supply to the outer retina, while in the inner retinal circulation, it is achieved by the general retinal circulation [34]. The neural retina is a thicker layer composed of cell layers responsible for transducing photons in electrical signals that posterior will pass through the optic nerve to the brain. The laminar architecture of the retina facilitates the processing of visual information. The neural retina is highly organized in (i) laminar structure and (ii) Müller glial cells. The laminar structure is formed by neuronal cells, such as retinal ganglion, amacrine, bipolar, horizontal cells, and rode and cone photoreceptors.

The posterior chamber of the eye is covered by a thick and fluid that looks similar to a gel, which is the vitreous humor that exists between the lens and the retina helping to maintain the structure of the eyeball. The viscosity of this fluid decreases with the advance of age, which allows the permeation of anterior aqueous humor into the posterior chamber, resulting in changes both in the retina and vitreous fluid. In some cases, if a significant tugging effect at the attachment point of retina occurs, detachment of the retina may happen [35].

The damage of the ocular surface leads to a weakening in corneal angiogenic processes and then stimulates the corneal neovascularization (CNV) and consequently leads to vision loss [36]. These alterations are usually correlated with aging as well as some ocular diseases, for example, age-related macular degeneration (AMD), glaucoma, and diabetic retinopathy (DR). The mostly used topical ocular formulations for the treatment of these diseases are ointments, gels, eye drops, and soft contact lenses [37]. 

NEs have been studied due to their safety and to improve the ophthalmic drug bioavailability with sustained drug release. In fact, the application of NEs in the eye can reduce significantly the side effects caused by the frequent administration of conventional methods, for example, intravitreal injections [38]. Cationic NEs represent an advancement in ocular drug delivery of poorly-water soluble drugs for several ocular diseases [39,40]. Due to their cationic character, upon ocular application, NEs increase the pre-corneal residence time by electrostatic interaction with the anionic ocular surface [41], resulting in the enhanced drug penetration through corneal tight junctions and increased drug bioavailability [42]. 

In the ocular administration of a drug, the major challenge is to achieve the desired therapeutical level and maintain this level at the target site because of the anatomy and physiology of the eye. The limited capacity of the cul-de-sac and the elimination of drugs from the lacrimal fluid are pre-corneal parameters that compromise the achievement of a therapeutical level. The cul-de-sac has a maximum capacity of 30 μL in humans, and it can be drastically reduced by the movement of the eyelid to its normal position [43]. The volume of eye drops administrated to the cul-de-sac can also be influenced by various pathological conditions, for example, allergic conditions and inflammation. These two pathological conditions decrease the capacity of the cul-de-sac and therefore are barriers against ocular drug delivery [44]. Two barriers against ocular drug delivery exist, and these can be classified as static or dynamic barriers. The anterior static barriers are composed by the (i) cornea, (ii) conjunctiva, (iii) blood aqueous barrier, and (iv) the efflux pumps [45]. The anterior dynamic barriers are (i) conjunctival lymph vessels, (ii) blood flow, (iii) tear drainage, and (iv) opposite directional flow of aqueous humor [46]. Posterior static barriers are (i) sclera, (ii) Bruch’s membrane, (iii) blood–retinal barrier, and (iv) efflux pumps [47]. The dynamic barriers of the posterior segment of the eye are (i) choroidal blood and (ii) lymphatic circulation [48]. The elimination of drugs from the lacrimal fluid occurs at the pre-corneal site due to the formulation drainage, lacrimation, and weak absorption in the conjunctiva [49]. 

The cornea is an impermeable physical barrier against mechanical and chemical damages and helps in the convergence of light in the retina [50]. The structure of the retina acts as a barrier against the absorption of drugs into the eye. The retina is organized in different layers, i.e., epithelium, stroma, and endothelium. In addition to the structural issues that influence the drug absorption, the molecular weight, and the charge of the drug, the degree of ionization and hydrophobicity also influence the permeability of drugs through the corneal layers [51]. The trans-corneal permeation is the principal pathway to the entrance of the drug into the aqueous humor via lacrimal fluid. 

Other ocular barriers that affect drugs absorption are the blood–ocular barriers. Blood–ocular barriers are responsible for maintaining a controlled environment in order to protect the eye from foreign composts, i.e., drugs, in the blood. Blood–ocular barriers include the blood–aqueous barrier (BAB) and the blood–retinal barrier (BRB). The BAB is present in the anterior part of the eye and blocks the entrance of different solutions in the intra-ocular environment [52]. To cross the BAB, the drugs must be lipophilic and of small molecular weight and can be more quickly removed from the anterior compartment than the hydrophilic and larger particles [53]. BRB is present in the posterior part of the eye and is constituted by endothelial cells and the RPE cells. This barrier blocks the entrance of toxic molecules and components of plasma in the retina; thus, it is able to limit the entrance of drugs systemically [54]. However, it has been postulated that these barriers can be crossed by lipophilic drugs when delivered by NEs [55]. Much more limited are the highly water-soluble drugs, as these cannot be formulated in o/w NEs [56].

## 3. Nanoemulsions for Ocular Delivery

Among the different types of NEs, o/w NEs have been an attractive solution for ocular drug delivery due to their characteristics. The more attractive characteristics of o/w NEs are (i) the presence of water as a continuous phase, which promotes easily the dilution with the physiological eye fluids (the tears), (ii) this type of NEs has the ability to entrap lipophilic drugs in the oily phase, and (iii) a deep penetration of the drug into ocular barriers is ensured by the increased retention time. The enhanced retention time is achieved by the reduction of the contact angle between the cornea and the formulation’s drops, increasing then the spreading coefficient and consequently the wettability and the retention time of administered NEs [44]. Surfactants used in the formulation of ocular delivery systems have been described as responsible to reduce the contact angle between the interface of the cornea and the applicated drops [44,57].

NEs are widely studied as a cost-effective formulation and non-invasive method due to their ability to improve drug bioavailability. Moreover, ophthalmic NEs have several advantages [9,44,58]: (i) prolonged pre-corneal retention time, (ii) high penetration ability, (iii) improved ocular bioavailability, (iv) enhanced drop drainage through the cornea and reproducible quantities of the drug in the eye if compared to gels or ointments, (v) the interface of lipid present in NEs to the lipid layer of tear film contributes to increasing the retention of ocular formulations in the conjunctival sac for a longer time, (vi) by using cationic NEs, it is possible to increase the drug residence time by electrostatic interactions with the anionic surface of the mucin of the cornea, consequently increasing the drug bioavailability in the eye. The interaction between the cationic NEs and the surface of mucin increases the residence time of NEs in the pre-corneal site; however, it is unlikely that the particles penetrate the cornea due to the bound made. In this case, it is expectable that the delivery of the drug occurs by passive diffusion over time.

The use of cationic surfactants in combination with anionic surfactants results in a synergistic effect highlighted by the low critical micelle concentration, enhanced surface activity, and increased tensioactivity. However, mixtures of cationic and anionic surfactants have a tendency to precipitate [59].

The eye contains three layers: a lipid layer, aqueous layer, and mucin layer. Each one has its function; the lipid layer is responsible for preventing the evaporation of water and allowing the conservation of the lubrification of the eye and the surface tension of the tear, the aqueous layer is dispersed homogenously in the entire eye surface, and the mucin layer is able to change the lipophilicity of the corneal epithelium [44]. The interaction of NEs with these layers assures the increased retention time and spreading the drop on the eye surface and consequently increasing the bioavailability of the entrapped drug. The use of surfactants in the formulation of NEs results in enhancing the wettability of the tear film and increasing the interaction of NEs with the mucin layer [44].

### 3.1. Methods of Preparation of NEs

To start the preparation of NEs, it is necessary to choose the components, i.e., (i) the oil used for the solubilization of lipophilic drugs, (ii) the appropriate surfactants, (iii) water to form the aqueous phase, and (iv) the co-surfactant (if needed to improve NEs stability) [12]. The preparation methods of NEs can be classified into two groups: the high-energy methods and the low-energy methods. The high-energy methods are high-pressure homogenization, microfluidization, and ultrasonication. The low-energy procedures are phase inversion emulsification processes (phase inversion temperatures and phase inversion composition) and the self-nanoemulsification method [44]. 

High-pressure homogenization is the most popular method, requiring homogeneous flow and high energy to generate smaller particle sizes. A hot pre-emulsion (macroemulsion) is passed through a small opening at high pressure and diverse forces (such as hydraulic shear, strong turbulence, and cavitation) are applied to create NEs. The pressure applied in this method is around 500 to 5000 psi [60,61]. The intense pressure promoted by the homogenizer induces deformation in the interface between the water and the oil phase of the emulsion, which promotes the breakdown of droplets into smaller ones [62]. In this process, it is necessary to have high energy input to create the interfacial area and, for that reason, the formation of NEs does not occur spontaneously.

In the microfluidization method, the macroemulsion is passed via micro-channels (microfluidizer) underneath high pressures and forces as shearing and cavitation generate NEs. The pressure applied in this method is around 500 to 20,000 psi [63]. This method can be used in the production of multiple NEs as well as in the production of dispersed phases with organized droplets sizes. Inside the microfluidizer, the solutions can be processed many times until the desired droplet size is achieved.

The ultrasonication method is one of the best ways to produce NEs on a laboratory scale. The agitation of molecules produced by the ultrasonic waves creates cavitation forces strong enough to break the macroemulsions down in NEs. Changing the power input and the time of ultrasonication, it is possible to reach the desired particle size [64]. The first proposed mechanism in ultrasonication was attributed to the generation of acoustic waves, which facilitates the dispersion of oil in the water phase. Another proposed mechanism results in the local generation of waves that helps the droplets of oil to break down to nano-sized droplets, which consequently contributes to them remaining stable over time [65].

In the phase inversion temperature method, with the change of temperature, surfactant spontaneous curvatures suffer inversion. Non-ionic surfactants, for example, polyoxyethylene-type surfactants, can suffer from dehydration of the polymer chain (with the increase in temperature) and consequently turn the surfactant more lipophilic and lead to alterations in the curvature of the surfactant, creating NEs [60]. If an anionic surfactant is used to produce NEs by this method, the spontaneous curvature of the droplet may suffer alteration by varying the temperature of the system. An o/w emulsion that was kept at low temperature, with the increasing of temperature will form a w/o emulsion with the addition of water obtaining the transitional phase inversion. On the other hand, by cooling a hot emulsion, the system achieves the point of zero spontaneous curvature and minimal surface tension and consequently forms finely dispersed oil droplets, obtaining the phase inversion of the system [66].

In the phase inversion composition method, with the change of the composition of the system, it is possible to get o/w NEs or w/o NEs with the addition of water to the oil surfactant or by adding oil to the water surfactant, respectively [60]. The initial volume of the oil used in the emulsion exceeds the quantity of water and with the addition of surfactants results in the reduction of droplets’ surface tension [67].

To produce NEs using the self-nanoemulsification method, hydrophilic surfactants or co-surfactants are added and maintained a low lipid content, which allows the self-assembly of surfactants and consequently creates o/w NEs [68]. This method is performed at room temperature and does not require any special equipment. Water is firstly added to the oily phase (containing the oil and surfactant) step by step at a determined temperature, and under stirring, the o/w NEs is generated. It is a spontaneous process; however, it depends on the (i) surfactant concentration and structure, (ii) viscosity (either interfacial and bulk), and (iii) interfacial tension.

After production by one of these methods, the prepared NEs should be quality-controlled with respect to various parameters, such as (i) particle size, (ii) polydispersity index, (iii) zeta potential, (iv) viscosity, (v) pH, (vi) encapsulation parameters, (vii) osmolality, (viii) stability, (ix) surface tension, (x) rheological properties, (xi) drug release, and (xii) toxicity [39].

### 3.2. Characterization of NEs

The characterization of NEs is essential to find the optimal formulation and to monitor the formulation properties over storage time. The observation of the visual appearance is essential to characterize the NEs and allows identifying any relevant changes. NEs’ appearance may range from transparent or semitransparent/translucent to milky white, depending on the droplet size, type, and concentration of surfactant/cosurfactant and oil. Under long-term storage, creaming can happen, which is characterized by a reversible separation of NEs into two phases [44]. The percentage transmittance (%T) can be studied using a UV spectrophotometer. Lower droplet size may promote translucent or transparence appearance (i.e., high values of transmittance) because the small droplet size allows higher transmission of the light [69].

The mean particle size and polydispersity index are obtained by dynamic light scattering (DLS) with Zetasizer devices. The droplet size should be between 20 and 500 nm for ocular administration. The droplet size of NEs may be reduced with the addition of surfactants that can work as penetration enhancers and as preservatives [70]. The polydispersity index is obtained by the ratio of the standard deviation to the mean particle size. Values close to 0 mean the presence of monodispersed NEs and the highest stability [6,71,72].

Other techniques to characterize the particle size are nowadays emerging, such as nanoparticle tracking analysis and multi-angle light scattering. Nanoparticle tracking analysis is a technique that is able to count and measure the size of nanoparticles below approximately 30 nm. Nanoparticle tracking analysis displays an accurate concentration of nanoparticles and size distributions unlike other techniques [73]. Multi-angle light scattering measures the size of nanoparticles dispersed in a solution and has high efficiency in the acquisition of particle concentration and diffusion coefficient. Then, these parameters are used in the Stokes–Einstein equation to calculate the nanoparticle size [74,75].

Zeta potential is an indirect measure of the electrical charge of the surface of droplets, which may also be used to estimate the physical stability of NEs. It is measured via the conversion of the electrophoretic mobility of molecules in an electrical field into zeta potential using the Helmholtz–Smoluchowski equation [76]. High values of zeta potential suggest repulsive forces between droplets that are responsible for stabilizing the system. For the ocular delivery of drugs, a zeta potential around +20 mv was described as enough to obtain optimal electrostatic contact between the NEs and the cornea surface. A zeta potential of NEs from +20 to +40 mv is enough to prolong the precorneal retention time [77]. The charge of the droplet surface offers bioadhesion on the surface of eye tissues and ensures the electrostatic repulsion between the droplets of NEs.

Taking into account the morphological appearance, NEs should be analyzed by electron microscopy approaches. NEs are spherical with a uniform particle size distribution if seen with transmission electron microscopy and atomic force microscopy [39]. Both microscopic techniques have size distribution results comparable to those obtained with DLS [39].

The droplet volume determines the quantity of drug that reaches the eye, and the surface tension is proportional to that volume. NEs for ocular delivery should also be analyzed by a thermostatically controlled tensiometer. A higher surface tension indicates lower tear film stability. Surface tensions lower than 35 mN/m might be painful and uncomfortable to the eye. The surface tension in the range of 40 and 50 mN/m is recommended for ocular drug delivery [78].

NEs have a low viscosity, which is important for ocular instillation. Rheological measurements are essential to reveal the viscosity values of formulations. The NEs viscosity can be changed, enhancing the quantity of oil, and with the addition of gelling agents (for example, polymers), the viscosity can be improved [39]. High values of viscosity increase the retention time of NEs by decreasing the frequency of drainage, which increases the bioavailability; however, these formulations are less tolerated. Otherwise, NEs with low viscosity allow a high acceptance [79]. The viscosity of ophthalmic formulations must be around 2–3 mPa/s [79]. Doshi et al. reported that NEs with high values of viscosity and low values of surface tension cause enhanced retention time [80].

The encapsulation parameters (e.g., loading capacity and encapsulation efficiency) of NEs are governed by the molecular weight, lipophilicity, and structure of the loaded drug [81]. Prior to the quantification of loaded drug into NEs, separation of the inner phase from the dispersed phase of nanoemulsions can be done using ultrafiltration, ultracentrifugation, gel filtration, and microdialysis methods [82]. To determine the loading capacity (*LC*) and encapsulation efficiency (*EE*), the following equations (Equations (1) and (2)) are used [83]:

(1) $$LC\%=\;\frac{W_{drug}-W_{S}}{W_{Oil}}\;\times \;100$$

(2) $$EE\%=\;\frac{W_{drug}-W_{S}}{W_{drug}}\;\times 100$$

where *W_drug_* is the mass of drug used to produce NEs, *W_Oil_* is the mass of oil of the inner phase of NEs, and *W_s_* is the mass of drug quantified in the supernatant (non-loaded).

In NEs for ocular delivery, the pH influences the irritability of formulations. The values of pH below 4 or higher than 10 are known to cause eye injury [84]. As known, the pH of formulations applied via ocular influence the drug permeation [85].

Another essential characteristic of NEs for ocular delivery is the osmolality of the formulation that is based on colligative properties (freezing and boiling point and vapor and osmotic pressure) [86]. NEs with osmolality less than 100 mOsm/kg or more than 640 mOsm/kg were described as eye irritants. The ability of the eye to restore the osmolality after the application of non-isotonic formulation depends on the droplet size and can take 1 to 2 min.

Formulations for ophthalmic use must be sterile, which means that the formulations need to be prepared in aseptic conditions or undergo sterilization of the final product. To sterilize ophthalmic formulations, autoclaving and aseptic filtration are the most used methods. The aseptic filtration is an efficient and simple method, but it can affect the particle size during the sterilization process due to the use of a 220 nm filter. The autoclavation uses high temperatures, which can change the components and physicochemical properties of the formulations [39,87]. To confirm the sterility of the formulations, and the suitability of the used sterilization technique, the formulations should be incubated during a period of 14 days to check the microbial growth on the media [88].

To evaluate the potentially harmful effects of some materials and newer NEs for ocular application, in vivo, in vitro, and ex vivo rabbit eye tests are commonly performed [89,90,91]. The in vivo Draize test is used to study the potential of irritation of formulations in rabbit eyes. The substance or formulation under study is applied in one albino rabbit’s eye and the effect promoted after application, such as chemosis, corneal opacity, effects on iris, and inflammation and redness of conjunctiva, is studied. Then, the outcome is classified according to a scoring system as (i) causing no irritation (no category), (ii) serious eye damage (category 1), or (iii) eye irritation (category 2). Category 2 can be subdivided as 2A if the effects are reversible or 2B if the results cause effects after 7 or more days [92].

Hen’s egg test-chorioallantoic membrane (HET-CAM) test is a study of the irritation potential of formulations for topical use (skin diseases) or to treat ocular conditions [93]. Since the CAM and the vascularized tissues of humans are similar, this test is usually recommended for the study of the irritant potential of formulations [79]. If the results of HET-CAM assay shows that the substances are not irritant to the eye, they can be instilled in the eye with safety [79].

For the further characterization of NEs, cytotoxicity assays (MTT assay and/or Alamar Blue assay) using ocular cell lines (for example, ARPE-19 cell line) should be made to ensure the ocular biocompatibility of NEs. 

The loaded drugs in ocular delivery systems will be transported through the ocular tissues barriers to reach the desired target site. There are several models to confirm the transcorneal permeability of ocular formulations [94]. Rabbit, rat, or mouse are the most commonly used in vivo models, epithelial cells cultures are the preferential in vitro models, while reconstructed cornea or cornea after excision are the recommended ex vivo assays [95]. In the ex vivo transcorneal permeation, the drugs loaded into NEs are transported across ocular barriers (cornea, conjunctiva, and sclera), and this assay studies their transcorneal permeability. Rabbit, cow, pig, goat, and sheep are ex vivo transcorneal permeation models [96]. There are some permeability chambers available to study the permeability of drug formulations. The vertical Franz diffusion cell, the modified Franz diffusion cell, the modified Ussing chamber, the horizontal perfusion cells, and the polycarbonate corneal perfusion chamber are some examples of these permeability chambers. These permeability tests provide information about the ex vivo permeation rate (apparent permeability and effective diffusion coefficients).

## 4. Formulating Nanoemulsions for Ocular Delivery 

Formulating NEs for ocular delivery requires the appropriate choice of oily and aqueous phases. The oily phase is usually based on oil, for example, medium-chain triglycerides, mineral oil, vegetable oil, and others. Additionally, the phase oil has the cationic agent and the surfactant. On the other hand, the aqueous phase is formed by another surfactant, i.e., a co-surfactant (such as poloxamers), an osmotic agent (to adjust the viscosity), and water [9]. The oil/lipid of the oil phase increases the solubility of lipophilic drugs and enhances the drug entrapment. The surfactant decreases the surface tension between the oil phase and the aqueous phase due to an increase in the miscibility of the oil and the aqueous phase [97]. The cationic agents contribute to increasing the surface charge of NEs droplets and may also behave as preservatives [98]. In the aqueous phase, the co-surfactant is used to reduce the interfacial tension and decrease the concentration of surfactant of the oil phase. The osmotic agent, as the name indicates, regulates osmolality [9].

### 4.1. Selection of Oil/Lipid

In the preparation of NEs, the drug loading is an important parameter and the solubility of poorly soluble drugs depends on the NEs constituents. To select the oils or lipids to use in the preparation, the solubility of the drug in the chosen lipid is the most important feature. Other relevant characteristics of the oil/lipid are viscosity, density, refractive index, and interfacial tension [99]. To obtain smaller droplet size NEs, it is essential to choose an oil with low interfacial tension and viscosity [100,101]. The medium- or long-chain triglyceride lipids are widely used as lipid phase in NEs to increase the bioavailability and solubility of lipophilic drugs [102].

Fernandes et al. studied used soybean oil as a vegetable oil that operated as an internal phase of NEs as lipid for ocular delivery [83,103]. The interfacial tension (oil and water) for soybean is 22.1 mN/m [104], its viscosity is 0.0405 Pa·s^−1^ at 30 °C and 0.0232 at 50 °C Pa·s^−1^ [105], and the density is 0.9082 and 0.9023 g/mL at 37 °C and 50 °C, respectively [106]. Soybean is a vegetable oil and results from a complex mixture of triglycerides. Soybean oil (C_11_H_9_N_3_O_2._Na) is composed of saturated fat, monounsaturated fat, and polyunsaturated fat. These fatty acids include linoleic, oleic, palmitic, linolenic, and stearic [107]. The characteristics of soybean oil make it a good option to be used as a pharmaceutical excipient in the formulation of non-irritating, biocompatible, and cost-effective NEs. This lipid has been used to formulate many formulations, for example, liposomes, microspheres, dry emulsions, micro and nanoemulsions, nanocapsules, and self-emulsifying systems [108]. To choose the lipid to formulate NEs for ocular delivery, it is important to take into account the solubility of the drug in this lipid. For example, in age-related macular degeneration, an ocular disease that affects millions of people in the world, triamcinolone acetonide has been described as an efficient drug to prevent the evolution of this disease [109]. To entrap triamcinolone acetonide in NEs with soybean oil, as an example, it is necessary to study the solubility of the drug in this lipid [83,103]. The solubility of triamcinolone acetonide is described as 95 ± 8 µg/mL in soybean oil and 4700 ± 300 µg/mL in Tween 80 (normally used as a non-ionic surfactant in formulations of ocular delivery) [110]. The triamcinolone acetonide has a logP = 2.53, which is normally considered lipophilic, although it is described to be soluble in vegetable oils and insoluble in mineral oils [111]. Other lipids that can be used in ocular NEs to increase the solubility of hydrophobic drugs are Castor oil, Lipoid S75, Lipoid E80, Oleic acid, Triacetin, Lipoid E-80, DOTAP, and Phospholipon^®^ 90H, among others [44].

### 4.2. Selection of Surfactant and Cationic Agent 

Due to a high volume of oil droplets, the interactions of droplets may lead to some instability phenomena, such as coalescence, creaming, phase inversion, and Ostwald ripening [2]. Coalescence is an irreversible process where two or more droplets suffer fusion during contact, forming a single droplet. Creaming occurs due to the density differences of two phases that constitute NEs. As the oily phase (disperse phase) has less density, it rises to the top, forming a more concentrated layer. Phase inversion is a phenomenon that happens when agitated o/w NEs revert to w/o NEs and vice versa. Ostwald ripening is a phenomenon characterized by the disappearance of small droplets due to their dissolution in the vicinity of the larger droplets, which grow in size. To inhibit these phenomena, it is necessary to use adequate surfactants because they have the ability to create the dispersion of two immiscible phases due to the decreasing of the interfacial tension of droplets, originating smaller droplets and reducing the droplets tendency to flow together when mixing and consequently increasing the stability of the created NEs [112]. 

The critical micellar concentration (CMC) of surfactants is the concentration from which micelles are spontaneously formed, and for that reason, the surfactant used in the formation of NEs should be used at this concentration. Above the CMC, the surfactants will create more micelles but hardly reduce the free energy of the system, which is a disadvantage as the lower free energy of the system at minimum concentration value makes NEs thermodynamically stable and, consequently, the separation of phases of the NEs occur [113]. Surfactants create the dispersion of two immiscible phases due to the reduction of the interfacial tension [112]. Surfactants that have high HLB increase corneal permeation. Surfactants with HLB values of 8–16 originate o/w NEs [114]. Surfactants can be divided into categories as zwitterionic, non-ionic, anionic, and cationic, taking into account their electrical properties. The non-ionic surfactants, such as Tween 80, have been considered harmless and can cause reversible alterations on the permeability of the ocular surface. Tween 80 is widely used in ocular formulations due to its safety and non-irritant effect on the eye and has an HLB of 15 [115]. 

**Figure 2:**
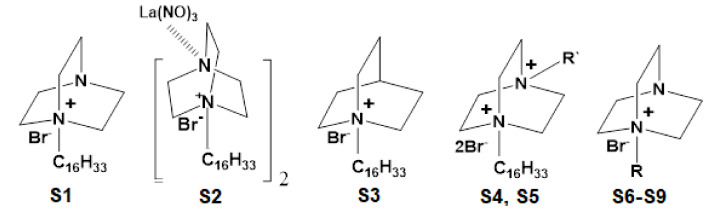
Structures of quaternary ammonium derivatives of quinuclidine (S3) and of DABCO derivatives (S1, S2, and S4 to S9 surfactants). Figure adapted from [121].

Tween 80 (C_64_H_124_O_26_) is described as a hydrophilic non-ionic surfactant; it is also used as a lubricant in eye drops, as an excipient that acts to stabilize aqueous formulations of drugs similar to disinfectant, and it has antimicrobial properties [116]. The use of cationic surfactants in combination with non-ionic surfactants revealed better colloidal stability [117]. The cationic surfactant creates an electrostatic repulsion between the droplets in order to stabilize the NEs [118]. The cationic quaternary ammoniums are preservatives that also act as surfactants and give to the NEs the cationic charge of droplets [9]. The cationic surfactants, such as quaternary ammonium compounds and zwitterionic surfactants, have been described with antimicrobial activity in relatively low concentrations [119]. Due to their characteristics (environmentally friendly, cleavable, and less toxic), quaternary ammonium derivatives of 1,4-diazabicyclo[2.2.2]octane (DABCO; C_6_H_12_N_2_) and of 1-azabicyclo[2.2.2]octane (quinuclidine; C_7_H_13_N) have been studied to be used widespread for applications in biotechnology [120]. Mono- and dicationic DABCO and quinuclidine surfactants as cationic surfactants (S1–S9) are represented in Figure 2. Other possibilities are the Tween 40 and 20, Span 20, Poloxamer 407, and Pluronic F68 [44]. Other cationic lipids such as DOTAP (N-(1-(2,3-dioleoyloxy)propyl)-N,N,N-trimethylammonium) chloride and DOPE (dioleoyl phosphatidylethanolamine) have been used in preservative-free cationic formulations for ocular administration [9].

### 4.3. Selection of Co-Surfactant

Similar to surfactants, co-surfactants are amphiphilic molecules with an active surface, small size, and work synergistically with the primary surfactants. Co-surfactants increase the fluidity of the interfacial film of droplets and decrease the concentration of surfactants needed, avoiding their potential toxicity [44].

The use of polyethylene glycol (PEG) as co-surfactant has been described to provide stealth properties to the formulations [122], being particularly suited for ophthalmic applications given its enhanced mucoadhesive properties. For that purpose, PEG-20 sorbitan monolaurate, monopalmitate, and monooleate have been proposed in the production of nanoemulsions [123].

Poloxamer 188 (a non-ionic surfactant) has been used as a co-surfactant in NEs [83,103]. Poloxamer 188 (C_8_H_18_O_3_) has been described elsewhere to create smaller particle size and narrower size distribution in emulsification [124]. Additionally, poloxamer 188 is a block linear copolymer characterized as having anti-thrombotic, with anti-inflammatory properties, and some rheologic activity as well as protective behavior anti oxidative stress [125]. NEs produced with Tween 80 and Poloxamer 188 after application revealed good spreading over the entire cornea–conjunctiva surface by gamma scintigraphy, which reveals the potential of combined use in formulations for ocular delivery [40].

### 4.4. Selection of Osmotic Agent

The osmotic agents in NEs have the function of maintaining the osmolality of the formulation. Glycerol (C_3_H_8_O_3_) is an osmotic agent widely used in NEs that works as a co-solvent in an aqueous solution and has the characteristic of changing the density, viscosity, and refractive index properties of the solutions [103]. It is also water soluble, and its addition to aqueous solution modifies the solubility of surfactants (whether ionic or non-ionic) and the optimum curvature of droplets [126]. In aqueous solutions, the presence of glycerol has an impact on the droplet size, being therefore expectable that small droplets are formed [127]. Increasing the concentration of glycerol in the aqueous phase of o/w NEs resulted in the decrease in the particle size when produced by high shear mixer or homogenization processes. On the other hand, in o/w NEs, the concentration of glycerol of about 1.5 wt % (low concentration) was related to increased storage stability over time [128]. Higher temperatures in NEs with high concentrations of glycerol in composition showed rapid droplet growth, i.e., instability due to increasing phenomena of coalescence or Ostwald ripening. At elevated temperatures, the dispersion in the presence of glycerol suffers modifications in the surfactant curve and in the solubility of the oil, increasing the risk of occurrence of these phenomena [126].

Synergistically with other components of o/w NEs, glycerol has been used for the ocular administration of drugs to treat some eye disorders, namely, dry eye syndrome and bacterial ocular infection, among others [129].

Fernandes el al., (2021) [83] successfully developed oil-in-water nanoemulsions with quaternary derivatives of 1,4-1,4-diazabicyclo[2.2.2]octane (DABCO) surfactants to entrap triamcinolone acetonide for ocular administration. Those formulations have in their composition Tween 80 as a non-ionic surfactant (that works together with the cationic surfactant to achieve better colloidal stability), soybean oil (internal phase), poloxamer 188 (co-surfactant), and glycerol (osmotic agent). The formulations produced in this study revealed the antifungal properties and encapsulation efficiency of triamcinolone acetonide ≈90% [83].

## 5. Conclusions

The increase in ocular absorption is a challenge in nanotechnology. Solid nanoparticles (either polymeric or lipid), liposomes, and nanoemulsions have been proposed for the delivery of drugs to ocular tissues. The permeation of drugs through ocular–blood barriers is the limiting factor that influences the bioavailability of drugs delivered through conventional ocular systems. Nanoemulsions provide a sustained drug release, as eye drop application is patient friendly and is possible to deliver poorly water-soluble drugs, increasing their bioavailability. The formulation factors of nanoemulsions, such as the choice of surfactants (e.g., cationic agents) and lipid composition, as well as the method of preparation (e.g., high pressure/high shear homogenization, ultrasonication) are instrumental to obtain the optimal formulation for ocular delivery. The optimization of processing parameters will contribute to reach a stable nanoemulsion formulation suitable for the delivery of drugs to treat and prevent eye disorders by means of sustained drug release, increasing the bioavailability of drug and biocompatibility.

## Data Availability

No new data were created or analyzed in this study. Data sharing is not applicable to this article.
